# Changes in insulin resistance following antidepressant treatment mediate response in major depressive disorder

**DOI:** 10.1177/02698811221132473

**Published:** 2022-11-15

**Authors:** Houman Rashidian, Mehala Subramaniapillai, Caroline Park, Orly Lipsitz, Hannah Zuckerman, Bing Cao, Yena Lee, Hartej Gill, Roger Nelson Rodrigues, Joshua D. Di Vincenzo, Michelle Iacobucci, Saja Jaberi, Joshua D. Rosenblat, Roger S. McIntyre, Rodrigo B. Mansur

**Affiliations:** 1 Mood Disorders Psychopharmacology Unit, University Health Network, Toronto, Ontario; 2 Department of Psychiatry, University of Toronto, Toronto, Ontario; 3 School of Psychology and Key Laboratory of Cognition and Personality (Ministry of Education), Southwest University, Chongqing, China; 4 Institute of Medical Science, University of Toronto, Toronto, Ontario; 5 Department of Pharmacology, University of Toronto, Toronto, Ontario; 6 Brain and Cognition Discovery Foundation, Toronto, Ontario

**Keywords:** Depression, major depressive disorder, antidepressant, insulin resistance, CRP, treatment response

## Abstract

**Background:**

Insulin resistance (IR) is a potential predictor of antidepressant treatment response.

**Aims:**

We assess changes in IR after antidepressant treatment and whether these changes have any effect on treatment response. Also, to see whether changes in IR mediates relationship between C-reactive protein (CRP) and antidepressant efficacy.

**Methods:**

This is a secondary analysis of an 8-week, open-label clinical trial with 95 adults experiencing a major depressive episode. Response to vortioxetine was measured using the Montgomery–Åsberg Depression Rating Scale (MADRS). Generalized estimating equation models were utilized for this intent-to-treat analysis.

**Results:**

When adjusted for age, sex, and body mass index, there was a significant increase in IR following treatment in the overall sample (p = 0.035). This finding was detected in treatment non-responders (p = 0.019), whereas it was not observed in responders (p = 0.329). Mediation analysis revealed that change in IR during treatment was responsible for change in MADRS as well as the relationship between baseline CRP and treatment response.

**Conclusions:**

Exacerbation of IR during antidepressant treatment mediated non-response. Conversely in treatment responders IR reduced. Like previous studies, baseline CRP moderated treatment response. This relationship was also mediated by changes in IR. These findings further elucidate the role of IR in terms of antidepressant response as well as potentially explain inflammation’s relationship with the latter.

## Introduction

Antidepressant medications are established treatment for major depressive disorder (MDD), despite response and remission rates remaining relatively low. According to the Sequenced Treatment Alternatives to Relieve Depression Trial, approximately 27% of individuals achieve full remission after the first antidepressant trial, with there being a greatly diminishing probability of remission with each subsequent trial. Even after utilizing four different agents, about one-third of patients remain in non-remission (Pigott, 2015). Suboptimal remission and recovery rates provide the basis for pursuing alternative, often less proven, treatment strategies to improve symptoms. This also includes developing predictors of treatment response. This will hopefully allow for more personalized mental healthcare ultimately leading to better outcomes in a more expedited fashion.

Insulin resistance (IR) has recently been uncovered as a potential predictor of treatment response. This refers to a state where insulin has an attenuated impact on glucose concentration and is a risk factor for the development of diabetes (Mantzoros, 2022). The current research group recently revealed decreased response to vortioxetine, a serotonergic antidepressant, in the context of IR, specifically with regard to the symptoms of anhedonia, subjective cognitive capabilities, and overall psychosocial function (Rashidian et al., 2021). This can potentially be explained by the fact that in the context of IR, glucose transport across the blood–brain barrier is decreased (Leonard and Wegener, 2019). There are also reports that both the production of tryptophan, which is the precursor to serotonin, and its ability to enter the brain are impaired (Jha et al., 2017). Thus, as a result of IR, there will potentially be less available serotonin available for the reuptake function of many antidepressants (McIntyre et al., 2005). In addition, disturbance of other monoamine systems, including dopamine and noradrenaline, is also included (Woo et al., 2016). Lastly, there is evidence that reward systems are impaired in the context of IR (Lyra e Silva et al., 2019) potentially providing the substrate for a decreased antidepressant response toward anhedonia.

Another potential reason for this finding can be IR’s relationship with the inflammatory marker C-reactive protein (CRP). Insulin dysregulation has been associated with systemic inflammation. Elevated CRP levels are correlated with the development of diabetes type II in both sexes (Grimble, 2002). More specifically, elevated CRP levels were linked to higher insulin and hemoglobin A1C (HbA1c) levels in both men and women and specifically increased glucose levels in women (Wu, 2002). This hyper-activation of the immune response is associated with IR and reduced pancreatic beta-cell function (McIntyre et al., 2005). IR itself is positively correlated with CRP levels even when controlling for body mass index (BMI; Ndumele et al., 2006). Baseline CRP levels are associated with more severe depression (Köhler-Forsberg et al., 2017) and treatment resistance (Zhang et al., 2019) especially to serotonergic medications, such as SSRIs (i.e. escitalopram, sertraline, fluoxetine, and paroxetine) and SNRIs (i.e. venlafaxine and duloxetine). This is in comparison to medications that also have an impact of the noradrenergic system. (Uher et al., 2014). In summary, both IR and CRP are associated with inadequate treatment response, and some of the proposed mechanisms of resistance are the same; both have deleterious effects on monoamines, including serotonin (Jha et al., 2017; Neurauter et al., 2008).

Conversely, whether IR changes post antidepressant treatment has been explored by various groups with differing outcomes. Okamura et al. (2000) detected an improvement in IR following successful treatment of depression with tricyclic antidepressants. This finding has not been confirmed by another group (Pyykkönen et al., 2011). Furthermore, whether this potential change in IR is what accounts for treatment response is something that has not been researched. This is what the current paper will attempt to uncover; to analyze whether IR changes in vivo during treatment can predict response. Lastly, further analyses looking into how these changes in IR can explain CRP’s role in treatment resistance will be completed. The goal is that we will continue to further elucidate the role of IR in depression and treatment response ultimately expanding our knowledge of this predictor of antidepressant response.

## Experimental procedures

### Subjects

Subjects were derived from an 8-week, open-label clinical trial that mainly assessed changes in cognitive function and performance using the THINC-integrated tool (THINC-it) in adults with MDD who received vortioxetine (10–20 mg flexibly dosed, *N* = 100) (ClinicalTrials.gov Identifier: NCT03053362). Subjects met the following eligibility criteria: (1) provided written informed consent; (2) male or female between 18 and 65 years old; (3) active diagnosis of a major depressive episode (MDE) as part of MDD as per Diagnostic and Statistical Manual, Fifth Edition criteria; (4) current MDE as confirmed by the Mini International Neuropsychiatric Interview 5.0.; (5) are managed via an outpatient psychiatric setting, (6) a Montgomery–Åsberg Depression Rating Scale (MADRS) score ⩾20 at screening and baseline; and (7) history of at least one previous MDE formally diagnosed by a healthcare provider or validated by previous treatment (e.g., guideline-informed pharmacotherapy and/or manual-based psychotherapy).

The following exclusion criteria applied: (a) benzodiazepines or alcohol use within 12 h of assessments; (b) abuse of marijuana; (c) physical, cognitive, or language impairments which adversely affect data derived from assessments; (d) formally diagnosed dyslexia or reading disability; (e) clinically significant learning disorder; (f) history of moderate or severe traumatic brain injury; (g) pregnancy and the postpartum period; and (h) other neurological diseases, or unstable systemic medical diseases. Subjects were at the Brain and Cognition Discovery Foundation in Toronto, Canada. Institutional review board consent was acquired before commencing the study.

### Assessments

The main outcome measure was sensitivity to change using the THINC-it tool. The secondary measure that is relevant to the current report is the MADRS. Response was defined as a 25% reduction in MADRS score. Metabolic data (Table 1) were obtained from all subjects at baseline and endpoint. BMI was measured using the equation BMI = weight (Kg)/height (meters)^2^, and overweight/obesity was defined as BMI ⩾25 kg/m^2^. Subjects also had whole blood samples collected after fasting for 12 h. Metabolic parameters were measured in a single laboratory with the same assay. IR in the basal state was calculated from fasting insulin and fasting plasma glucose using the HOMA2 calculator (http://www.dtu.ox.ac.uk) (Levy et al., 1998).

**Table 1.** Descriptive statistics of the ITT sample.

	MDD (*N* = 95)
Age (years), mean (SD)	38.85 (12.55)
Sex (female), *n*(%)	63 (66.3)
Age at onset (years), mean (SD)	20.24 (10.96)
Number of previous depressive episodes, median (IQR)	6.00 (3–20)
Duration of current MDE (months), median (IQR)	8.00 (5–24)
History of psychiatric hospitalization, *n*(%)	19 (20.0)
MADRS (total score), mean (SD)	31.94 (7.46)
IPAQ (total MET-minutes per week), median (IQR)	810.00 (297–1746)
BMI, mean (SD), normal range	28.55 (6.88), 18.5–24.9
Obesity, *n*(%)	37 (38.9)
MAP (mm/Hg), mean (SD), normal range	92.03 (11.02), 70–100
Fasting glucose (mmol/L), mean (SD), normal range	5.50 (1.29), 3.9–5.6
Fasting insulin (pmol/L), mean (SD)	69.37 (51.28)
HOMA-IR, mean (SD)	1.32 (0.97)
Total Cholesterol (mmol/L), mean (SD), normal range	4.99 (1.05), <5.2
CRP (mg/L), mean (SD), normal range	4.02 (6.46), <10 mg/L

BMI: body mass index; CRP: C-reactive protein; HOMA-IR: homeostatic model assessment of insulin resistance; IPAQ: international physical activity questionnaire metabolic equivalent task; IQR: interquartile range; ITT: intent-to-treat; MADRS: Montgomery–Åsberg Depression Rating Scale; MAP: mean arterial pressure; MDE: major depressive episode; SD: standard deviation.

### Statistical analysis

All subjects were included as this was an intent-to-treat analysis. Since the distribution of metabolic and clinical outcomes were non-normal, generalized estimating equation models were utilized. For analyses with metabolic variables as outcomes, the best fit was found with gamma distribution and an independent covariance structure. For analyses with MADRS as the outcome, negative binomial models with log link specification and independent covariance structure were selected. The independent variables were time (as a categorical variable), and all models were adjusted for age and sex. Moderation of baseline variables (e.g., time by baseline CRP interaction) was assessed in separate models. Due to the nonlinearity of the models, the estimated β coefficients were transformed into rate ratio estimates. To assess mediation, we used the PROCESS macro (Hayes, 2013) in SPSS v24.0. Bootstrapping with 5000 resamples was performed to determine bias-corrected (asymmetric) 95% confidence intervals (CIs) for indirect effects (Preacher and Hayes, 2008).

## Results

A total of 100 subjects were included; 95 had laboratorial data and were included in the analysis. In all, 79 (83.15%) of those completed the 8-week follow-up. Baseline socio-demographic, clinical, and laboratorial characteristics are described in Table 1. After adjustment for age and sex, there was a significant effect of time on IR (χ^2^ = 4.431, *p* = 0.035), indicating a significant increase in IR following treatment in the overall sample. There was also an effect of time on high-density lipoprotein (HDL) levels (χ^2^ = 4.293, *p* = 0.038), which also increased. There were no effects of time on BMI, mean arterial pressure, Hb1Ac, triglycerides, low-density lipoprotein, and CRP (all *p* values > 0.05). The effect of time on IR remained significant after further adjustments for BMI (χ^2^ = 4.322, *p* = 0.038) and HDL (χ^2^ = 8.820, *p* = 0.003).

**Figure 1.**
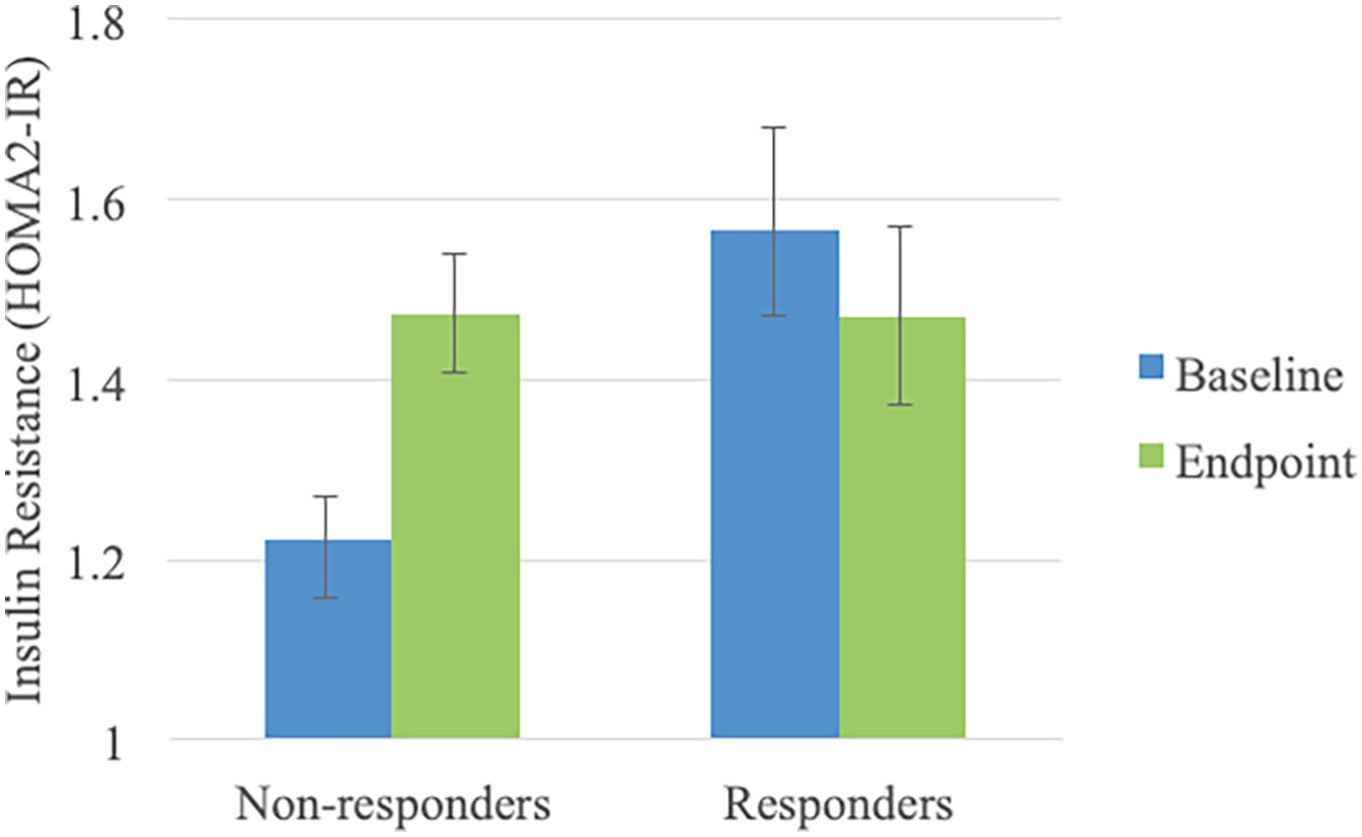
Baseline and endpoint estimated means and standard errors of insulin resistance (HOMA2-IR) values, according to treatment response. HOMA-IR: homeostatic model assessment of insulin resistance.

At the study endpoint, there was a significant correlation between percent change in MADRS scores and IR (*r* = 0.379, *p* = 0.001), indicating that reduction in depressive symptoms was associated with a reduction in IR measures. There were no associations between changes in IR and changes in other metabolic parameters (all *p* values > 0.05). Conversely, treatment response moderated the observed changes in IR (time by response interaction: χ^2^ = 6.748, *p* = 0.009); treatment non-responders had a significant increase in IR (mean difference 0.24, standard error (SE): 0.10, *p* = 0.019), which was not observed in treatment responders (mean difference −0.09, SE: 0.09, *p* = 0.329) (Figure 1). To further understand the relationship between changes in IR and treatment response, mediation models were used. We observed that the differences in response between responders and non-responders were partially mediated by changes in IR and there was a small but significant indirect effect of change in IR on change in MADRS scores. There was no exposure–mediator interaction (*p* = 0.100). The overall model, adjusted for age and sex, was significant (F4,70 = 40.812, *R*^2^ = 0.700, *p* < 0.001). The significance of the indirect effect was not modified by further adjustment for baseline IR (−0.031, 95% CI −0.070; −0.001) or baseline MADRS (−0.033, 95% CI −0.070; −0.006), indicating that the finding is unlikely to be the result of mean reversal.

**Figure 2.**
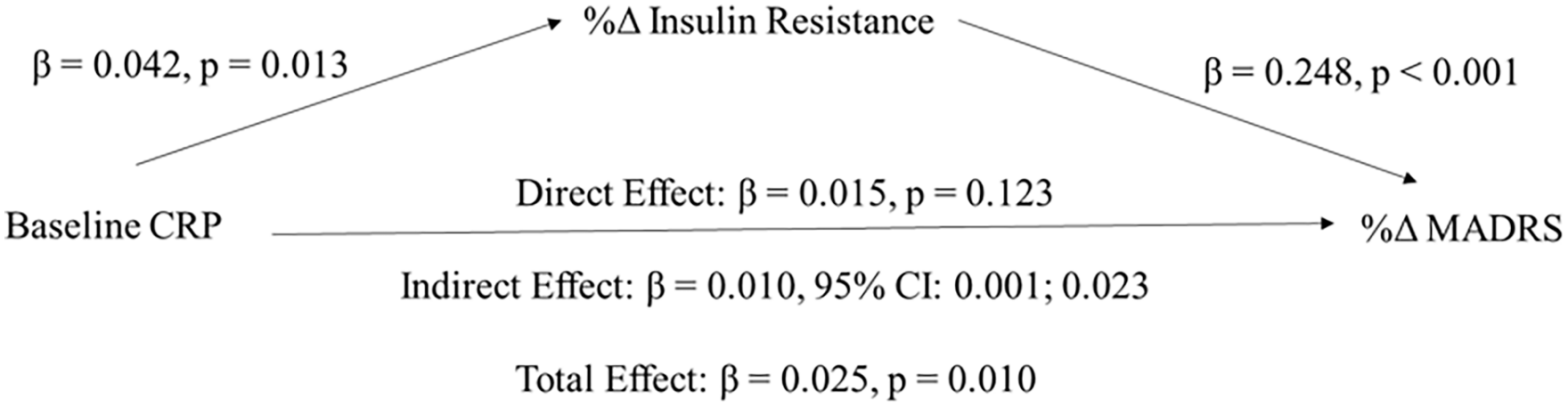
Unstandardized regression coefficients from a bootstrap-mediation analysis indicating that percentage changes in insulin resistance fully mediated the effects of baseline CRP on percentage changes in depressive symptoms severity following treatment. CRP: C-reactive protein.

Predictors of response were subsequently assessed. As previously documented, baseline CRP moderated treatment response (time by baseline CRP interaction: χ^2^ = 8.882, *p* = 0.012); higher baseline CRP was associated with poorer antidepressant response. Conversely, baseline CRP also moderated changes in IR (time by baseline CRP interaction: χ^2^ = 5.431, *p* = 0.020); higher baseline CRP was associated with increases in IR following treatment. No other baseline metabolic variable moderated changes in MADRS scores and IR values (all *p* values>0.05). Mediation model indicated that the effect of baseline CRP on treatment response was fully mediated by changes in IR (Figure 2). There was no exposure–mediator interaction (*p* = 0.183). The overall model, adjusted for age and sex and baseline IR (as baseline IR and CRP were correlated, *r* = 0.314, *p* = 0.002), was significant (F5,69 = 5.717, *R*^2^ = 0.210, *p*<0.001).

## Discussion

The results of our analyses indicate that increased IR during our study mediated treatment non-response. The opposite was detected in treatment responders where IR decreased at the endpoint of therapy. Baseline CRP significantly moderated treatment response: higher baseline CRP levels were associated with poorer outcomes. Furthermore, it was found that the relationship between baseline CRP and depression outcomes was mediated by changes in IR; higher baseline CRP led to increases in IR which ultimately led to non-response.

As previously discussed, higher baseline IR is associated with more severe depression (Lyra e Silva et al., 2019; Singh et al., 2019) and reduced response to treatment (Rashidian et al., 2021). To our knowledge, our results are the first to show reduced insulin sensitivity during the process of non-response. This in conjunction with baseline IR both appear to play a role in treatment response or lack thereof. This falls in line with previous work related to inflammation; a recently completed meta-analysis synthesized outcomes from longitudinal studies that followed inflammatory markers at baseline and during treatment. This meta-analysis found significant decreases in tumor necrosis factor-alpha (TNF-α) levels in treatment responders, while TNF-α remained elevated in treatment non-responders (Strawbridge et al., 2015). It is not surprising that a similar pattern was found in IR, as it has close ties to inflammatory states (Grimble, 2002).

As already reviewed, CRP is a predictor of poor response to antidepressant therapy (Zhang et al., 2019), particularly serotonergic agents (Uher et al., 2014). The current study found a similar response pattern with vortioxetine, where higher baseline CRP levels were associated with attenuated response to vortioxetine. A notable finding was that higher CRP levels at treatment onset modified the changes in IR; higher inflammation was linked with augmented IR increases following treatment and it was this increase which explained treatment non-response. Mechanistically, this can be explained as CRP can lead to cytokine activation and bind to damaged membranes of vascular cells, activating complement, and thrombogenic systems. The resulting vascular inflammation can subsequently lead to IR, although the mechanism is not yet fully understood (Gelaye et al., 2010). Furthermore, previous work assessing CRP and IR in the context of cardiometabolic risk factors in young individuals revealed that an increased duration of IR was required before the association of CRP and other metabolic risk factors with IR became clinically salient (Moran et al., 2005). In other words, decreased sensitivity to insulin may underlie the negative impacts CRP has on antidepressant response rate.

Several limitations of the current study should be noted. First, this was a post-hoc analysis where the relationship between IR and CRP was not the primary outcome. This was also an open-label uncontrolled study, which may have biased the assessment of our outcomes. Vortioxetine was the only antidepressant prescribed to subjects, and thus extrapolation to other serotonergic agents is limited and warrants further research. Furthermore, metabolic and inflammatory laboratorial data were obtained from a single center and were limited in size. Lastly, we did not exclude individuals with acute illness or infections, as this was not the primary aim of the original study. As such, subjects may have been predisposed to transiently elevated CRP values that were not accounted for. Similarly, physical comorbidities, many of which are associated with elevated CRP, are themselves a predictor of poor response (Dodd and Berk, 2004) and thus may have confounded our results.

The present analysis presents preliminary data, which reveals that an exacerbation of IR mediates treatment non-response. Furthermore, CRP’s well-known impacts on treatment response in depression seem to further be explained by the changes in insulin regulation. Overall, this points to a more important and mechanistic role of IR within the interplay of depression and its treatments. Future studies should try confirming these current initial findings as well as exploring whether these effects are found with other serotonergic medications as well as agents that act on other monoamine systems.
